# SNP-based bulk segregant analysis revealed disease resistance QTLs associated with northern corn leaf blight in maize

**DOI:** 10.3389/fgene.2022.1038948

**Published:** 2022-11-25

**Authors:** Ruining Zhai, Aihua Huang, Runxiu Mo, Chenglin Zou, Xinxing Wei, Meng Yang, Hua Tan, Kaijian Huang, Jie Qin

**Affiliations:** ^1^ Maize Research Institute, Guangxi Academy of Agricultural Sciences, Nanning, Guangxi, China; ^2^ Guangxi Academy of Agricultural Sciences, Nanning, Guangxi, China

**Keywords:** northern corn leaf blight, disease resistance, bulk segregant analysis, SNP, QTL

## Abstract

Maize (*Zea mays* L.) is the most important food security crop worldwide. Northern corn leaf blight (NCLB), caused by *Exserohilum turcicum*, severely reduces production causing millions of dollars in losses worldwide. Therefore, this study aimed to identify significant QTLs associated with NCLB by utilizing next-generation sequencing-based bulked-segregant analysis (BSA). Parental lines GML71 (resistant) and Gui A10341 (susceptible) were used to develop segregating population F_2_. Two bulks with 30 plants each were further selected from the segregating population for sequencing along with the parental lines. High throughput sequencing data was used for BSA. We identified 10 QTLs on Chr 1, Chr 2, Chr 3, and Chr 5 with 265 non-synonymous SNPs. Moreover, based on annotation information, we identified 27 candidate genes in the QTL regions. The candidate genes associated with disease resistance include *AATP1*, *At4g24790*, *STICHEL-like 2*, *BI O 3-BIO1*, *ZAR1*, *SECA2*, *ABCG25*, *LECRK54*, *MKK7*, *MKK9*, *RLK902*, and *DEAD-box ATP-dependent RNA helicase*. The annotation information suggested their involvement in disease resistance-related pathways, including protein phosphorylation, cytoplasmic vesicle, protein serine/threonine kinase activity, and ATP binding pathways. Our study provides a substantial addition to the available information regarding QTLs associated with NCLB, and further functional verification of identified candidate genes can broaden the scope of understanding the NCLB resistance mechanism in maize.

## Introduction

Maize (*Zea mays* L.) is a significant food security crop worldwide, fulfilling human and animal nutrition requirements (Tilman et al., 2011; Stevens and Madani, 2016; Cole et al., 2018). The gradual increase in global population is rapidly impacting food demand, as are the environmental impacts on crops (Tilman et al., 2011). China is among the leading maize producer and consumers, with a 21% share in global maize production (Yang et al., 2019a). The adversities of climate change tend to increase the intensity of both biotic and abiotic factors influencing crop yield (Li et al., 2017; Vaughan et al., 2018; Onyekachi et al., 2019). Maize is prone to multiple pathogens inducing rust, northern corn leaf blight, southern corn leaf blight, and leaf streak, which negatively affect photosynthesis activities. Without effective disease management strategies, the yield losses can reach about 70% (Romero, 2016). Moreover, host resistance is considered an effective long-term control compared to temporary chemical control with potential hazards of chemical pollution (Johnson, 1981; Venter, 2007; Singh et al., 2016; Nazir et al., 2021a).

Genetic control in crops can be categorized as qualitative and quantitative resistance. Qualitative resistance is induced by major resistance genes and is generally genotype-specific with less durable resistance (Parlevliet, 2002; Darino et al., 2016; Galiano-Carneiro and Miedaner, 2017). Several studies have demonstrated the identification of key resistance genes against rust and northern corn leaf blight in maize, including *Rp* and *Ht1*, *Ht2*, *Ht3*, *HtM*, *and HtN* genes (Darino et al., 2016; Galiano-Carneiro and Miedaner, 2017). The effectiveness of qualitative resistance tends to decline with the rapid evolution of pathogens (Ji et al., 2010). However, quantitative resistance controlled by several genes is considered the most durable against biotic stresses (Miedaner et al., 2020). Quantitative resistance is predominant in maize, with durable resistance to several races of pathogens (Wisser et al., 2006; Yang et al., 2017; Navarro et al., 2021). Therefore, it is pertinent to explore the genetic variation for effective disease control to attain sustainable crop production.

Northern corn leaf blight (NCLB) is a frequently occurring disease in maize in humid temperate and tropical climates (Ceballos et al., 1991). Minute chlorotic flecks generally characterize NCLB initially, and grey-green elliptical lesions form irregular areas of dead tissues at the mature stage (Wise, 2011). *Exserohilum turcicum, a* filamentous hemibiotrophic fungus*,* is the causal pathogen for NCLB in maize (Wu et al., 2014). Several studies have characterized the evolution and mode of infection and the physiological impact of *E. turcicum* in maize (Leonard and Suggs, 1974; Chung et al., 2010; Ohm et al., 2012; Kotze et al., 2019; Silveira et al., 2019). A study by Wu et al., (Wu et al., 2014), identified and characterized several miRNAs in response to *E. turcicum* in maize. Similarly, another study by Zang et al., (Zang et al., 2020), identified ERF transcriptions factor regulating responses against *E. turcicum* in maize. Moreover, ERF genes are involved in several critical pathways, such as ethylene, salicylic acid, and jasmonic acid regulating disease resistance (Xu et al., 2007; Xie et al., 2019).

Climate change, cultivation patterns, susceptible genotypes, and strong pathogenicity of *E. turcicum* are the major reasons for the increased occurrence of NCLB (Xia et al., 2020; Ma et al., 2022). Improving genetic makeup to develop more resistant cultivars is necessary to overcome food security challenges. With the advancement in technology, several methods have been extensively utilized to identify and characterize QTLs (quantitative trait loci) associated with biotic and abiotic phenomena. Moreover, next-generation sequencing technologies have accelerated the conventional breeding for robust identification of key biological regulators associated with the specific trait (He et al., 2020; Nazir et al., 2020; Nazir et al., 2021b). However, bulk segregant analysis (BSA) remains popular for its effectiveness and robustness in identifying key differences between two distinct characters (Karim et al., 2020).

The present study focused on the identification and characterization of major QTLs associated with NCLB in maize by utilizing a segregating population (F_2_) developed from GML71 (resistant parent) and Gui A10341 (susceptible parent). The genomic DNA from 30 plants of each category resistant and susceptible (identified after inoculation) was pooled for downstream analysis. QTLs with candidate genes related to Corn spot resistance were identified and annotated by combining the phenotype with genotype to provide a genetic basis for NCLB disease in two contrasting parental genotypes using Next-generation sequencing (NGS)-based bulked-segregant analysis (BSA).

## Materials and methods

### Plant material

Segregating population F_2_ was developed using two parental lines, GML71 and Gui A10341. GML71 is resistant to NCLB, while Gui A10341 is highly susceptible to NCLB. In spring 2019, two parental lines were planted in the Mingyang base and crossed to obtain F_1_ and sampled in two replicates to extract DNA during the jointing period. In the autumn of 2019, F_1_ was planted, and 283 fruit ears were obtained. In the spring of 2020, F_2_ was planted with a total of 283 individuals, and the disease resistance was investigated after artificial inoculation of *E. turcicum* (Pass.) LeonardetSuggs. Samples from 30 high-sensitive and 30 resistant lines were collected and bulked for each extreme for resequencing and BSA (bulk segregant analysis).

### Pathogenicity tests using colonized sorghum grains as inoculum

White sorghum grains, soaked overnight in a 250 ml flask, were used for inoculum preparation. After 24 h, water was drained, and flasks were covered with cotton and aluminum foils and autoclaved at 121°C. Spores of *E. turcicum* isolate NGIB16-13 were harvested and adjusted to a concentration of 105 spores ml^−1^. Flasks containing the sterilized sorghum were aseptically inoculated with 4 ml spore suspension and incubated at room temperature for 5 days. After incubation, the colonized grains were stored in a refrigerator (4°C) and later used for the inoculations.

The whorl of 21-day-old maize plants was inoculated with three colonized sorghum grains and covered with clean nylon bags for 48 h. Control plants were inoculated with sterile, non-inoculated sorghum. Four plants from two pots were evaluated for disease severity using the 1 to 9 (where 1 indicates no occurrence of disease, and nine indicates 100% incidence of disease) scale described earlier (Galiano-Carneiro et al., 2021).

### DNA extraction, library construction, and sequencing

Allele frequency estimates in BSA analysis depend on the variation in segregant samples and sequencing technology. The variation due to segregant samples can be minimized by either increasing the number of segregants or bulk size (Magwene et al., 2011). In this study, a total of 60 young leaves of F_2_ individuals (30 from resistance and susceptible pool each) along with two parental lines were collected, and genomic DNA was extracted using the cetyltrimethylammonium bromide (CTAB) method. The isolated DNA was quantified using a Qubit2.0 Fluorometer (Thermo, CA, United States). Equal amounts of DNA from the resistant and susceptible individuals were mixed to prepare the resistant pool (named D3) and susceptible pool (named D4). The DNA extracted from parental lines was also prepared for library construction, named D1 (GML71) and D2 (Gui A10341). The samples were sonicated to generate ∼350-bp fragments using an M220 instrument (Covaris, Woburn, MA, United States). Then DNA fragments were end-polished, A-tailed, and ligated with the adapter for PCR amplification (Lybaybio, Tianjin, China). Finally, PCR products were purified and analyzed for size distribution by an Agilent2100 Bioanalyzer and quantified using real-time PCR. Libraries, after quality inspection, were loaded onto an Illumina sequencing platform (Illumina, Inc., San Diego, CA, United States) for Hiseq X10 PE150 sequencing.

### Data processing and analysis

Sequencing data were aligned to previously published genome B73 using BWA (Li, 2013). Before alignment, raw data were processed for quality control by removing reads with ≥10% unidentified nucleotides, Phred quality <5, and not aligned >10reads. GATK pipeline was used for SNP calling (do et al., 2016). The read-depth information for the SNP index was estimated according to the method of Takagi et al. (Takagi et al., 2013) using a sliding window. The difference in the SNP index of the two pools was calculated as the delta SNP index.

We used some commonly used BSA analyses with multiple testing using SNP-index, Euclidean Distance (ED) (de la Fuente Cantó and Vigouroux, 2022), and G-statistics (Magwene et al., 2011). If there is an apparent major QTL that controls the corresponding trait, the significant effect intervals obtained by each method should not differ much; If there is no apparent major effect site, the results obtained by each analysis method may be different. SNP index (∆SNP) refers to the subtraction of the alternate allele frequency value of the low bulk from the high bulk (∆SNP) (Takagi et al., 2013). G-statistics takes advantage of a log-likelihood statistic LOD between allele frequencies (Zhang et al., 2019), while Hill et al., predicted Euclidean distance (ED) between two vectors defined by the frequencies of the alternate and the reference alleles in the high and low bulks to identify the region of interest (Hill et al., 2013). We estimated the overlapping QTLs from each test for quality results, and only common QTLs were carried out for further analysis.

Based on ∆SNP, G-statistics, and ED, we narrowed down the candidate region associated with NCLB resistance, and to further understand the genes in candidate regions; we conducted GO enrichment analysis on genes (http://www.geneontology.org/).

### Candidate gene identification and qRT-PCR based verification

Candidate genes concerning NCLB were further screened from each QTL interval using GO and KEGG enrichment information. Candidate genes with GO terms associated with disease resistance were subjected to qRT-PCR to check their expression pattern in contrasting genotypes (resistant and susceptible). Total RNA was extracted from fresh leaves and roots using TRIzol^®^ Plus RNA Purification Kit (Invitrogen, CA) based on the manufacturer’s instructions. Approximately 1 μg RNA was reversely synthesized into cDNA using the iScriptTM Synthesis Kit (Quanta BioSciences, MD). The qRT-PCR was carried out in an Eppendorf real-time PCR equipment using a 5 μl cDNA template (diluted 1/100), 5 μl primers (2.4 M), and 10 μl SYBR green mixture (Promega, Madison, WI). Histone 3 was used as the internal control, and the relative expression levels of the *ORP* gene were calculated by the 2^−ΔΔCt^ method (Livak and Schmittgen, 2001).

## Results

### Genotyping and SNP filtering

**FIGURE 1:**
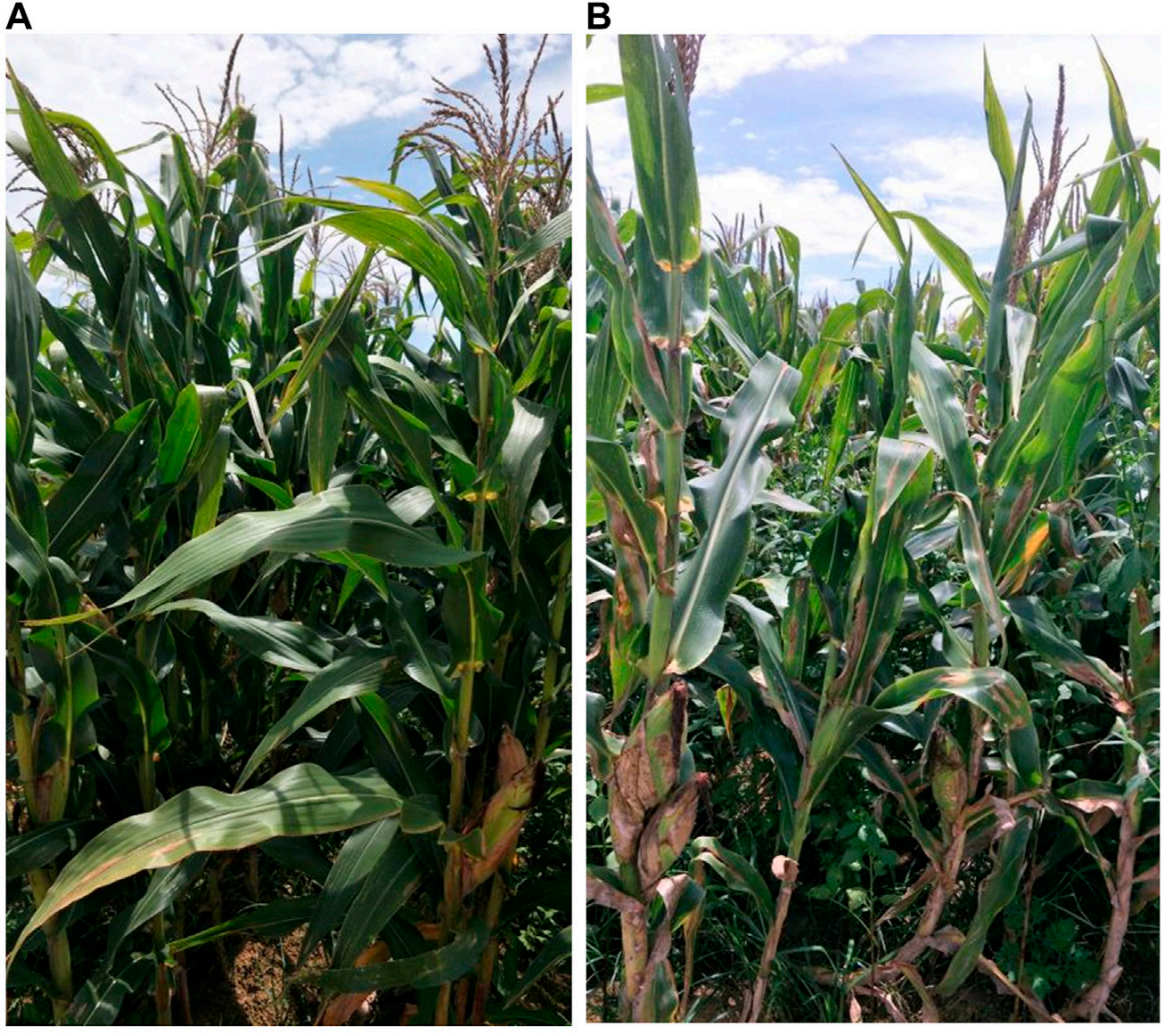
Morphological description of parental lines **(A)** GML71 (Resistant) and **(B)** Gui A10341 (susceptible).

To comprehend NCLB regulatory mechanisms in maize, we utilized two advanced lines, GML71 and Gui A10341 (Mo et al., 2019), to develop an F_2_ segregating population. GML71 is resistant, while Gui A10341 is highly susceptible to NCLB (Figure 1). The segregating population consisted of 283 individuals and was further categorized for both extremes, i.e., resistant and susceptible. The bulked samples from both resistant (D3) and susceptible (D4) groups, along with parental lines (GML71 as D1 and Gui A10341 as D2), were re-sequenced using Hiseq x10 technology. A total of 37.56 Gb of clean data was obtained, with 95.84% of reads showing an average score of Q20 and 89.78% of the reads showing an average score of Q30 (Supplementary Table S1). The GC contents in D1, D2, D3, and D4 were 47.94%, 47.98%, 55.82%, and 53.09%, respectively. The sequenced samples were aligned to the reference genome B73. The alignment details have been provided in Supplementary Table S2.

### BSA mapping

**FIGURE 2:**
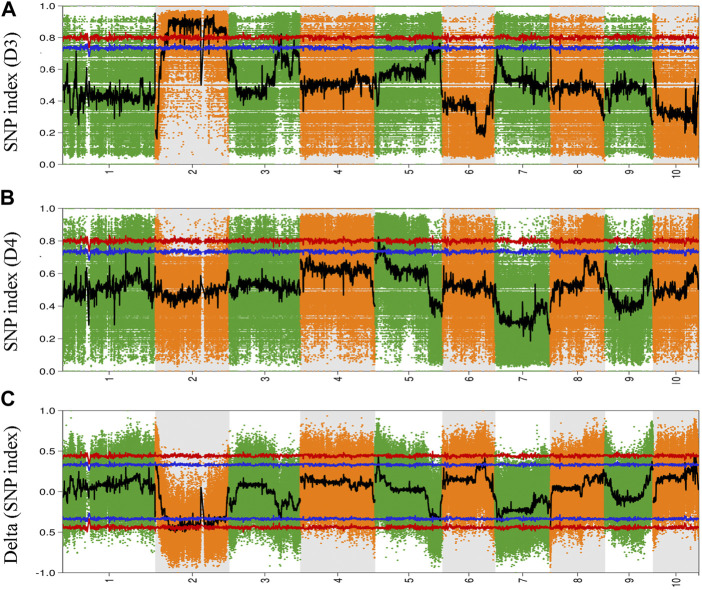
Genome-wide distribution of SNP-index in **(A)** D3 bulk and **(B)** D4 bulk **(C)** Delta (SNP-index) between De bulk and D4 bulk. The abscissa is the chromosome name, the colored dots represent the calculated SNP-index (or ΔSNP-index value, and the black line is the fitted SNP-index or ΔSNP-index value. The red line represents the threshold line with a confidence level of 0.99; the blue line represents the threshold line with a confidence level of 0.95.

**FIGURE 3:**
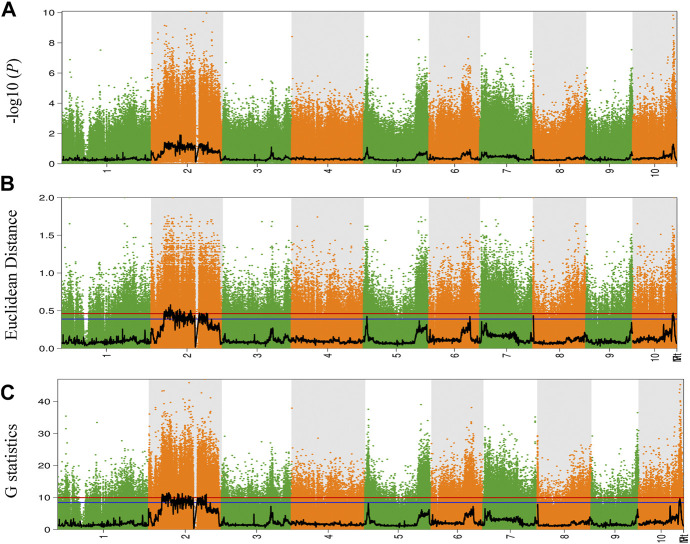
The results of the **(A)** -log10(*P*), **(B)** Euclidean distance (ED), and **(C)** G-statistics algorithm. The abscissa is the chromosome name; the colored dots represent the calculated SNP-index index (or ΔSNP-index value, and the black line is the fitted SNP-index or ΔSNP-index value. The red line represents the threshold line with a confidence level of 0.99; the blue line represents the threshold line with a confidence level of 0.95.

To explore and identify the molecular markers associated with NCLB disease resistance in maize, SNP-indices of each locus in D3 and D4 bulks were estimated using quality-filtered SNPs. The high-quality SNPs were classified as having a quality score ≥100 with a read depth ≥10. The average SNP-index in D3 and D4 bulks were estimated using a 2-Mb genomic interval with 10-Kb sliding windows. The Δ (SNP-index) between D3 and D4 bulks was also estimated and plotted for all the chromosomes of the maize genome (Figure 2). Similarly, Fisher’s exact test was also performed for the D3 and D4 bulks at each SNP locus, and the average *p*-values for SNPs located in each sliding window were calculated and log-transformed (Figure 3). Moreover, Euclidean distance and G statistics were also performed to further verify identified peak signals, and both produced similar QTL detection plots (Figures 3B,C). G-statistics indicate the allelic effect on quantitative traits (de la Fuente Cantó and Vigouroux, 2022).

Based on the Δ (SNP-index) statistics, we identified multiple peaks on Chr 2, Chr 5, Chr 6, Chr 9, and Chr 11. However, the most significant variation was observed on Chr 2 (Figure 2C). Therefore, we considered regions on Chr 2 as a hotspot for NCLB control in maize. To identify the significant QTLs and genes present in the QTLs, we set criteria of 99% confidence interval and identified a total of eight QTLs associated with D3 bulk on Chr 1, 2, and 3 (Supplementary Table S3). We named these QTLs as Q-Chr-number. For instance, Q2-1 denotes the first QTL identified on Chr 2. Similarly, we identified two QTLs on Chr five associated with D4 bulk (Supplementary Table S4). Q2-3, Q2-4, and Q2-5 were identified with interval size greater than 24 Mb, while the remaining QTLs were identified with less than 5.5 Mb intervals. Detailed SNPs statistics have been provided in Supplementary Tables S1, S2. Q1-1 spanned a 2.012 Mb region on chromosome 1 starting from 3,05,020,001 to 307,041,717. The region contained 48 genes. Five QTLs Q2-1, Q2-2, Q2-3, Q2-4, and Q2-5 were 5.45Mb, 3.09Mb, 24.01Mb, 85.19Mb, and 73.01Mb, respectively (Table 1).

**TABLE 1:** Statistics for QTLs identified with a 99% confidence interval.

No	QTLs	Chr	Start (Mb)	End (Mb)	Interval (Mb)	Genes	SNPs
1	Q1-1	1	305.02	307.04	2.02	48	21
2	Q2-1	2	30.82	36.28	5.46	131	1,332
3	Q2-2	2	36.92	40.02	3.10	74	739
4	Q2-3	2	40.66	64.68	24.02	405	9,027
5	Q2-4	2	65.22	150.42	85.20	716	37,138
6	Q2-5	2	162.06	235.08	73.02	1844	49,230
7	Q3-1	3	164.44	166.84	2.40	19	167
8	Q3-2	3	171.06	172.74	1.68	36	1,588
9	Q5-1	5	10.48	12.90	2.42	82	2,405
10	Q5-2	5	13.52	15.20	1.68	69	1,519

### Candidate genes and enrichment analysis

We further characterized each QTL and identified genes residing in the QTL regions. A total of 48, 131, 74, 405, 716, 1844, 19, 36, 82, and 69 genes were identified in Q1-1, Q2-1, Q2-2, Q2-3, Q2-4, Q2-5, Q3-1, Q3-2, Q5-1, and Q5-2, respectively. The identified genes were further subjected to GO-term ontology and KEGG pathways analysis (Supplementary Figures S1, S2). GO terms, including Signal transduction, hormone regulation, and pathogenesis, were enriched in D3 bulk (Supplementary Figure S1), while phosphorylation, phosphorus metabolic process, protein phosphorylation, and signal transduction were enriched in D4 bulk (Supplementary Figure S2). KEGG pathways enriched in D3 bulk were identified as MAPK signaling, sulfur metabolism, ABC transporters, and phenylalanine metabolism, while MAPK signaling, phosphonate metabolism, signal transduction, and sugar metabolism were enriched in D4 bulk. We identified 99,481 and 3,924 significant SNPs in D3 and D4 bulk, respectively. The identified SNPs were further screened for non-synonymous SNPs as candidate sites. 250 non-synonymous and frameshift SNPs were identified in D3 bulk, and 15 non-synonymous SNPs were identified in D4 bulk.

After SNP screening, we identified 6, 6, 30, 52, 151, 2, 4, 11, and 4 non-synonymous SNPs in Q2-1, Q2-2, Q2-3, Q2-4, Q2-5, Q3-1, Q3-2, Q5-1, and Q5-2, respectively. *ZmWAK-RLK1* gene has been previously characterized for involvement in NCLB in maize (Hurni et al., 2015; Yang et al., 2017; Yang et al., 2019b; Yang et al., 2021a). The annotation information regarding *ZmWAK-RLK1* suggested involvement in several pathways, including protein phosphorylation, cytoplasmic vesicle, protein serine/threonine kinase activity, and ATP binding pathway in this study (Li et al., 2020). Therefore, we further screened genes associated with non-synonymous SNPs using annotation information and identified 27 genes on chromosomes 2 and 5 (Table 2). Q2-5 contains 14 annotated genes associated with pathways related to protein phosphorylation, cytoplasmic vesicle, protein serine/threonine kinase activity, and ATP binding pathway. Q2-5 genes associated with disease resistance encode *cyclin11*, *receptor-like kinase*, *Putative leucyl-tRNA synthetase*, *Protein kinase superfamily protein*, *leucine-rich repeat receptor-like serine*, *Protein kinase superfamily protein*, *LRR receptor-like serine/threonine-protein kinase*, *Serine/threonine-protein kinase UCNL*, *ATP-dependent DNA helicase*, *Probable inactive receptor kinase*, *CHROMATIN REMODELING 5*, *ATP-dependent DNA helicase chloroplastic*, *Wall-associated kinase 2-like protein*, and *ATP-dependent DNA helicase*. *Mitogen-activated protein kinase 9*, *Mitogen-activated protein kinase 9*, *Atypical receptor-like kinase MARK*, *and DEAD-box ATP-dependent RNA helicase 21* were identified in Q5-1. Moreover, QTLs Q2-4, Q2-5, and Q5-1 were identified with multiple genes associated with disease resistance pathways; therefore, we consider these QTLs as candidates for further functional verification. Further molecular insight into the functions of genes associated with candidate QTLs can provide a comprehensive overview of quantitative disease resistance against NCLB in maize.

**TABLE 2:** Distribution of candidate genes associated with resistance to NCLB on Chr 2, and five

chr	QTLs	Start	End	GeneID	Symbol	Description
2	Q2-1	30,820,001	36,280,000	Zm00001d003209	*AATP1*	*AAA-ATPase ASD mitochondrial*
2	Q2-2	36,920,001	40,020,000	Zm00001d003266	*At4g24790*	*Protein STICHEL-like 2*
2	Q2-3	40,660,001	64,680,000	Zm00001d003430	*EMB2768*	*Tyrosine--tRNA ligase chloroplastic*
2	Q2-4	65,220,001	1,50,420,000	Zm00001d004592	*BI O 3-BIO1*	*-*
2	Q2-4	65,220,001	1,50,420,000	Zm00001d004635	*ZAR1*	*Leucine-rich repeat protein kinase family protein*
2	Q2-4	65,220,001	1,50,420,000	Zm00001d004707	*SECA2*	*thylakoid assembly1*
2	Q2-4	65,220,001	1,50,420,000	Zm00001d004762	*ABCG25*	*ABC transporter G family member 25*
2	Q2-4	65,220,001	1,50,420,000	Zm00001d004905	*LECRK54*	*OSJNBa0027H06.10 protein*
2	Q2-5	162,060,001	235,080,000	Zm00001d005293	*CYCD3-2*	*cyclin11*
2	Q2-5	162,060,001	235,080,000	Zm00001d005298	*At4g37250*	*receptor-like kinase*
2	Q2-5	162,060,001	235,080,000	Zm00001d005558	*At1g09620*	*Putative leucyl-tRNA synthetase*
2	Q2-5	162,060,001	235,080,000	Zm00001d005682	*PBL28*	*Protein kinase superfamily protein*
2	Q2-5	162,060,001	235,080,000	Zm00001d005776	*XA21*	*leucine-rich repeat receptor-like serine*
2	Q2-5	162,060,001	235,080,000	Zm00001d005829	*STY17*	*Protein kinase superfamily protein*
2	Q2-5	162,060,001	235,080,000	Zm00001d005986	*RGI3*	*LRR receptor-like serine/threonine-protein kinase*
2	Q2-5	162,060,001	235,080,000	Zm00001d006017	*UCNL*	*Serine/threonine-protein kinase UCNL*
2	Q2-5	162,060,001	235,080,000	Zm00001d006695	*--*	*ATP-dependent DNA helicase*
2	Q2-5	162,060,001	235,080,000	Zm00001d006917	*At5g10020*	*Probable inactive receptor kinase*
2	Q2-5	162,060,001	235,080,000	Zm00001d007089	*CHR5*	*Protein CHROMATIN REMODELING 5*
2	Q2-5	162,060,001	235,080,000	Zm00001d007417	*At3g02060*	*ATP-dependent DNA helicase chloroplastic*
2	Q2-5	162,060,001	235,080,000	Zm00001d007474	*WAK3*	*protein; Wall-associated kinase 2-like protein*
2	Q2-5	162,060,001	235,080,000	Zm00001d007562	*ESD4*	*ATP-dependent DNA helicase*
5	Q5-1	10,480,001	12,900,000	Zm00001d013418	*MKK7*	*Mitogen-activated protein kinase 9*
5	Q5-1	10,480,001	12,900,000	Zm00001d013423	*MKK9*	*Mitogen-activated protein kinase 9*
5	Q5-1	10,480,001	12,900,000	Zm00001d013430	*RLK902*	*Atypical receptor-like kinase MARK*
5	Q5-1	10,480,001	12,900,000	Zm00001d013453	*Os03g0708600*	*DEAD-box ATP-dependent RNA helicase 21*
5	Q5-2	13,520,001	15,200,000	Zm00001d013527	*CPK9*	*Calcium-dependent protein kinase 30*

### Expression profile of candidate genes

**FIGURE 4:**
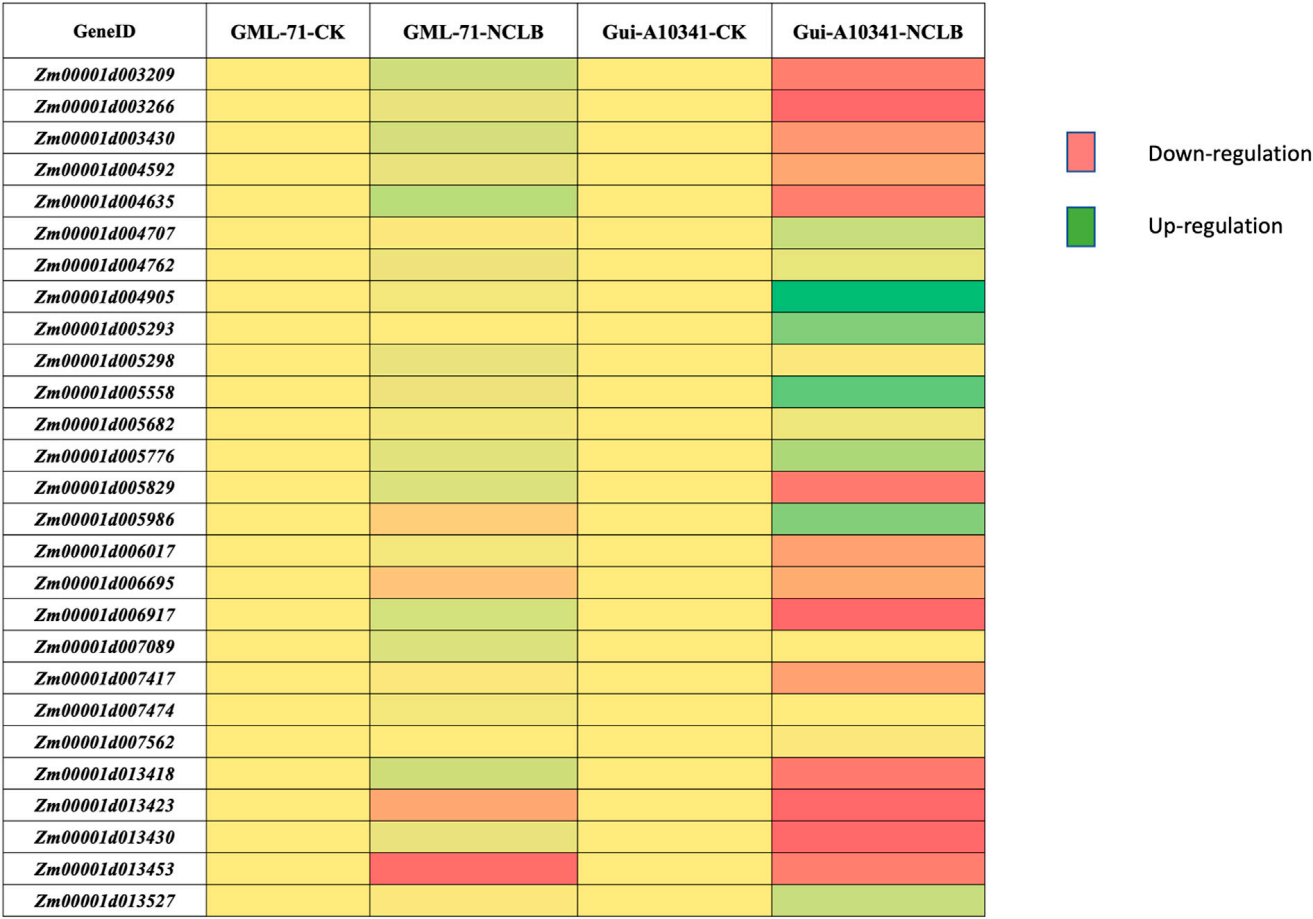
Expression profile of candidate genes in two contrasting parental genotypes, GML-71 and Gui A10341. The expression pattern was estimated before (check = CK) and after NCLB inoculation (NCLB) in both genotypes.

To understand the regulation patterns of candidate genes in two contrasting parental genotypes (GML71 is resistant, while Gui A10341), we performed qRT-PCR for all the candidate genes. The primers for each candidate gene are listed in Supplementary Table S5. The expression profile of each parent was considerably different when the expression was compared after the inoculation of NCLB. Among 27 candidate genes, 14 depicted up-regulated expression patterns under disease inoculation in tolerant genotype Gui A10341, while six showed down-regulated expression patterns (Figure 4). *Zm00001d003209, Zm00001d003266, Zm00001d003430, Zm00001d004592, Zm00001d004635, Zm00001d005829, Zm00001d006017, Zm00001d006695, Zm00001d006917, Zm00001d007417, Zm00001d013418, Zm00001d013423,* and *Zm00001d013430* were among the 14 up-regulated genes in resistant genotype. Zm00001d013453In comparison, susceptible genotype GML71 was observed with up-regulated expression pattern of only four genes (*Zm00001d005986*, *Zm00001d006695*, *Zm00001d013423*, *Zm00001d013453*). The differential expression pattern after inoculation is highly suggestive that these genes are excellent candidates for further function verification and use in breeding programs for NCLB resistance.

## Discussion

NCLB, caused by *Exserohilum turcicum*, is a frequently occurring foliar disease of maize, threatening more than 50% yield losses in tropic and sub-tropic environments (Blandino et al., 2012). Resistant cultivars are the effective control for NCLB (Technow et al., 2013). Multiple studies have depicted chemical control as an effective measure for NCLB control (Blandino et al., 2012; Zhang et al., 2013; Camera et al., 2019; De Rossi et al., 2022). However, chemical and biological control increase the cost of production along with environmental hazards (Alabouvette et al., 2006; Fry, 2012; Hirooka and Ishii, 2013). Recent advances in omics with high-density molecular markers have enabled plant breeders to successfully understand and manipulate molecular markers to the genetic architecture of modern cultivars (Collard and Mackill, 2008; Nazir et al., 2021b; Sarfraz et al., 2021). The present study successfully utilized bulk segregant analysis to identify molecular markers associated with NCLB.

Previously reports suggested a complex genetic architecture of NCLB with multiple QTLs spanning multiple chromosomes (Wisser et al., 2006; Poland et al., 2011; Van Inghelandt et al., 2012). Poland *et. al*., identified 29 QTLs related to NCLB throughout the genome (Poland et al., 2011). However, each QTL depicted a minor effect in contribution towards NCLB resistance. Recently, with the advancement of technology, the BSA method supported by genomic and transcriptomic data has been proven to be a robust and efficient platform identification of molecular markers and associated genes related to a particular trait in several crops, including rice, maize, brassica, wheat, and cotton (Mansfeld and Grumet, 2018; Gyawali et al., 2019; Mu et al., 2019; Yang et al., 2021b; Wang et al., 2021). In the present study we did not perform a phenotypic evaluation on the complete F_2_ population. This would have allowed a chi-square analysis to predict the number of QTLs responsible for the variation of disease score in the population. We instead utilized resistance and susceptible bulks as plant materials and identified 10 QTLs potentially associated with the NCLB resistance mechanism in maize. These QTLs were identified on Chr 1, Chr 2, Chr 3, and Chr 5. A similar work by Li *et*. *al*., (Li et al., 2020), reported 502 non-synonymous SNPs in six QTLs related to NCLB on Chr eight and identified seven putative candidate genes involved in NCLB control. We identified 27 candidate genes in seven QTLs combined with SNP-index and annotation information.

Both qualitative and quantitative inheritance patterns of NCLB resistance have been reported in maize (Galiano-Carneiro and Miedaner, 2017). Identification and utilization of NCLB resistance controlled by a single gene, such as *Ht1*, *Ht2*, and *Ht3,* is ineffective for long-term resistance. Several physiological phenomena have been confirmed associated with the functions of these genes, including inhibition of chlorotic spots, epiphytotic control, and signal transduction (Welz and Geiger, 2000; Hurni et al., 2015; Wang et al., 2018). Moreover, several biological pathways associated with disease resistance in maize have been identified, such as protein phosphorylation, cytoplasmic vesicle, protein serine/threonine kinase activity, and ATP binding pathway (Li et al., 2020). Quantitative resistance with the cumulative effect of several genes is necessary for durable resistance against NCLB (Li et al., 2020). Three genes, *AATP1* (*AAA-ATPase ASD mitochondrial*), *At4g24790* (*STICHEL-like 2*), *EMB2768* (*Tyrosine--tRNA ligase chloroplastic*), were identified as candidate genes in Q1-1, Q1-2, and Q3-2, respectively. A previous study by Liu et al., (Liu et al., 2020), reported activation of *OsAAA-ATPase1* during blast infection in rice, suggesting a key role of *OsAAA-ATPase1* salicylic acid-mediated defense responses against fungus *M. oryzae*. Moreover, *ATPase* genes are known for their active role in hypersensitive response (HR) toward plant pathogens (Dmitriev et al., 2017; Weiß and Winkelmann, 2017; Waititu et al., 2021). Nitric oxide (NO) regulates developmental and stress-mediated responses in plants by activating NO-signaling pathways (León, 2022) associated with Tyrosine-protein. Similarly, *STICHEL-like 2* has been reported to be involved in response to biotic and abiotic stress responses (Hu et al., 2015; Wang et al., 2015; Hejri et al., 2021).

We identified four candidate genes in the QTL Q2-4, including *BI O 3-BIO1*, *ZAR1*, *SECA2*, *ABCG25*, *and LECRK54*. *BI O 3-BIO1* regulates biotin synthesis, an essential cofactor for enzymes catalyzing carboxylation, decarboxylation, and transcarboxylation reactions (Wang et al., 2020). Functional characterization of bio2 mutant in *Arabidopsis thaliana* suggested biotin decreased ROS (reactive oxygen species) accumulation under stress conditions (Wang et al., 2020). *ZAR1-RKS1* complex, through direct interaction with *RKS1*, triggers *ZAR1* activation and disease resistance in *A. thaliana* (Wang et al., 2019a). Several reports suggest a resistance response mediated by *ZAR1* (Lewis et al., 2010; Wang et al., 2019b; Hu et al., 2020; Chen et al., 2021). For instance, Wang et al., (Wang et al., 2019b), characterized the *ZAR1-RKS1* complex, which causes terminal cell death and mediates rapid stress-induced transcriptional activation of defense genes in plants.

Characterization of Q2-5 identified 12 genes associated with disease resistance response. These genes included *At4g37250*, *At1g09620*, *PBL28*, *XA21*, *STY17*, *RGI3*, *UCNL*, *At5g10020*, *CHR5*, *At3g02060*, *WAK3*, and *ESD4*. While, *MKK7*, *MKK9, RLK902*, *Os03g0708600*, and *CPK9* were identified as candidate genes from two QTLs on Chr 5. The annotation information suggested the involvement of these genes in disease resistance responses. Numerous QTL studies have reported QTLs associated with NCLB in maize. In the present study, the resolution was significantly improved by using high-density SNP markers, and the improved resolution can facilitate a comparison of our results with disease-associated QTL and genes reported previously. For example, Poland et al., (Poland et al., 2011), identified four QTLs on Chr 5 with one putative candidate gene *GRMZM2G024612* associated with NCLB resistance. Our results comprehend quantitative resistance response towards NCLB by identifying several QTLs spanning three chromosomes with multiple genes associated with the NCLB resistance mechanism. Another study by Chen et al., (Chen et al., 2016), identified a stable QTL on chromosome 8 with one putative candidate gene. Xie et al., (Xia et al., 2020), reported five QTLs associated with NCLB resistance on chromosomes 1, 2, 4, 8, and 9. Another genomic region important for NCLB resistance was identified in chromosome 8, where two associated genes (Ht2 and Htn1) were identified (Zaitlin et al., 1992; Hurni et al., 2015).

We identified ten QTLs on Chr 1, Chr 2, Chr 3, and Chr 5 with a 99% confidence interval associated with NCLB resistance. Moreover, we screened 265 non-synonymous SNP-containing genes and narrowed them down to 27 candidate genes with differential expression patterns in NCLB contrasting genotypes (susceptible and resistant). Our study provides a genetic basis for quantitative disease resistance against NCLB in maize. Further functional characterization of candidate genes based on the provided information can yield significant insights into the NCLB resistance mechanisms in maize.

## Data Availability

The raw sequencing data can be found in NCBI SRA under the project number PRJNA875291 (https://www.ncbi.nlm.nih.gov/bioproject/?term=PRJNA875291).
