# Supplementary figures and images for: SNP-based bulk segregant analysis revealed disease resistance QTLs associated with northern corn leaf blight in maize
Source: Front Genet. 2022 Nov 25;13:1038948. doi: 10.3389/fgene.2022.1038948 (PMC9732028; doi:10.3389/fgene.2022.1038948)

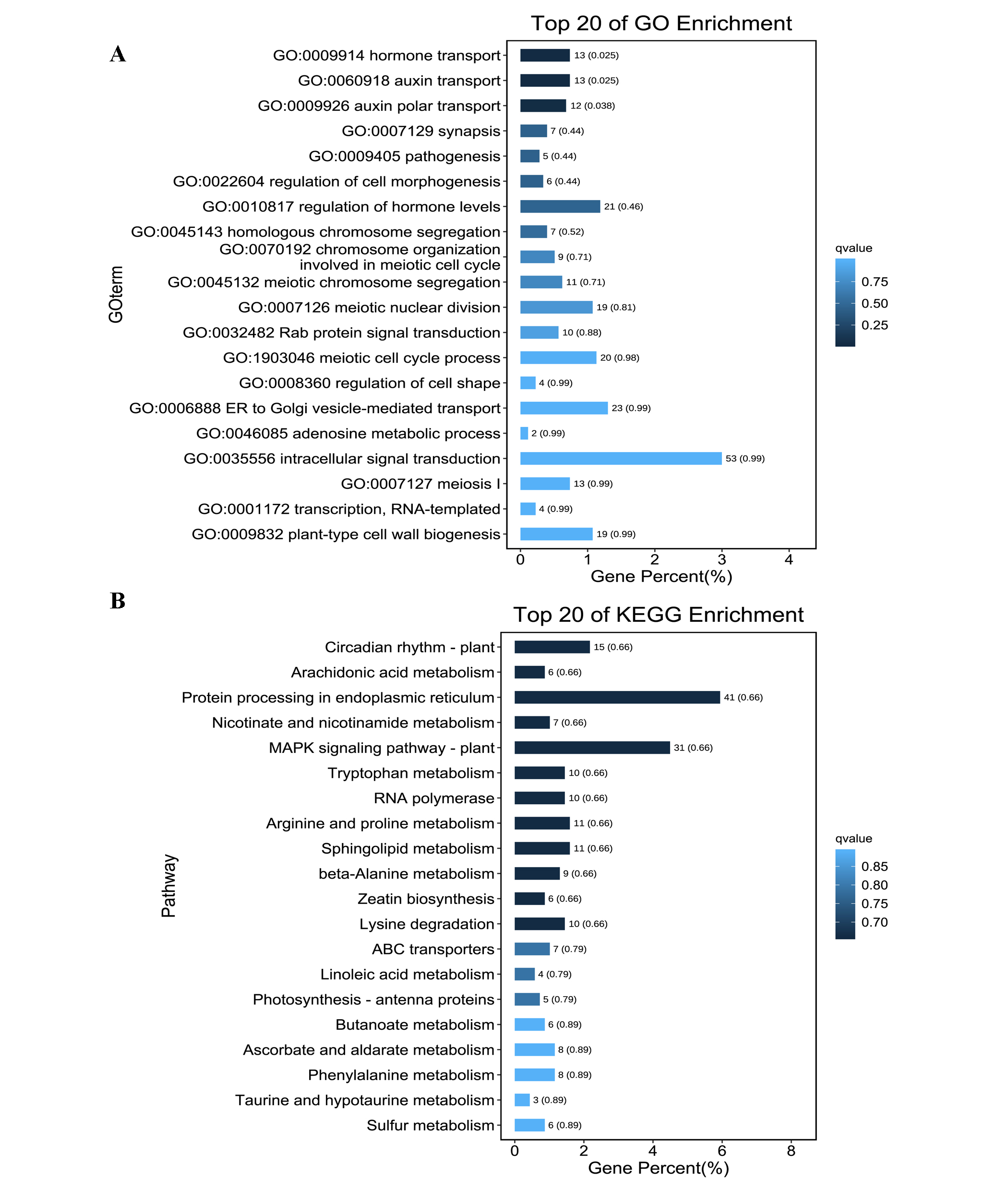

Supplement: Supplementary file 3 [file Image1.JPEG]

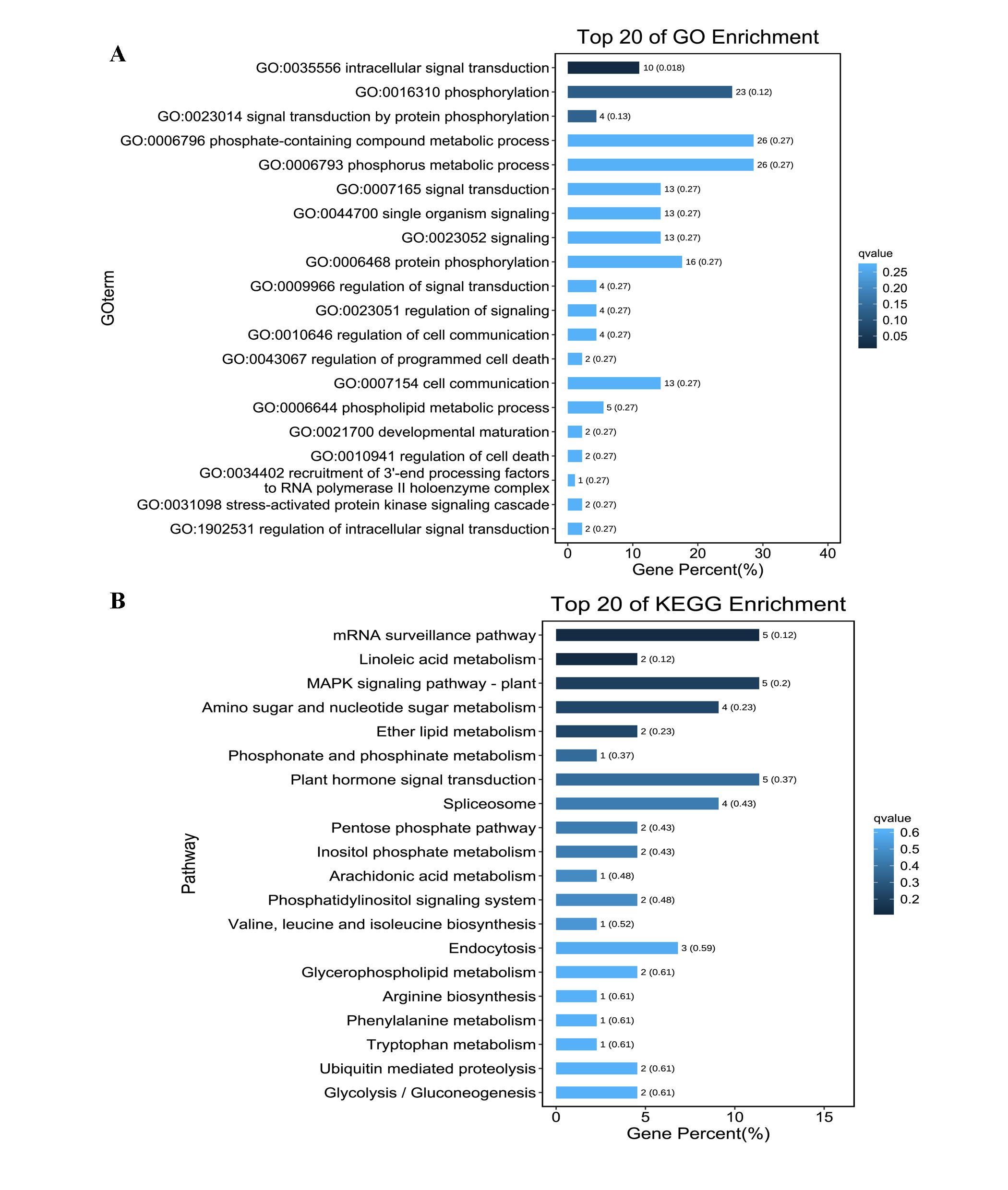

Supplement: Supplementary file 4 [file Image2.JPEG]
